# Does Wuhan Need to be in Lockdown during the Chinese Lunar New Year?

**DOI:** 10.3390/ijerph17031002

**Published:** 2020-02-05

**Authors:** Jing Wu, Michelle Gamber, Wenjie Sun

**Affiliations:** 1 Department of Infectious Diseases, SKLG, School of Life Sciences, Huashan Hospital, Fudan University, Shanghai 200040, China; 2 School of Health Professions, Division of Public Health, Shenandoah University, Winchester, VA 22601, USA; 3 The Second Affiliated Hospital of Fujian Traditional Chinese Medical University, Fuzhou 350003, China; 4 Robert Stempel College of Public Health and Social Work, Florida International University, Miami, FL 33174, USA

On 23 January 2020, the government of China announced a lockdown of all public transportation departing from Wuhan, including airports, trains, and buses. It is the first time in the history of Wuhan to have such a lockdown [1]. Wuhan is home to 11 million people and is considered the epicenter for a novel strain (nCoV) of Coronavirus (CoV), that has not previously been identified in humans. 

This current crisis, and subsequent lockdown, raises an important question—is a transportation lockdown in Wuhan necessary to control Wuhan pneumonia? Of importance to take note, January 23 is the Traditional China Lunar Mouse New Year (like Thanksgiving in the USA), where family members from other cities need to go home to celebrate the New Year. The Chinese Lunar New Year is the most important time for families to come together. Usually, this spring festival lasts for 7 days, during which Chinese citizens visit their relatives and friends. After the Traditional Lunar New Year, people return to their cities and resume work. Both the transportation to and visiting of family and relatives during this celebration period increase the likelihood and risk of transmission of respiratory disease. 

Although the WHO did not formally list China as a dangerous area for Wuhan pneumonia, Thailand, Japan, USA, and Singapore have already reported imported cases from China [2]. Due to the incubation period of the 2019-nCoV at around 14 days, there might be another outbreak peaking around 6 February 2020, a result of potentially infected people leaving Wuhan before or on 23 January 2020 by private transportation means. Those infected people might not have had symptoms at that time, and therefore could have spread the virus. 

The challenge of controlling Wuhan pneumonia caused by 2019-nCoV is to identify the original source of Wuhan pneumonia. Until now, it is unknown which animal is the reservoir for the coronavirus [3]. Huanan wholesale seafood market in Wuhan city has been suspected as the source of the outbreak, yet this has not been confirmed [3,4]. In 2013, when there was the Asian lineage avian influenza A(H7N9) virus outbreak, a suspected poultry market was shut down. Likewise, the wholesale seafood market has been shut down since 1 January 2020. 

A significant difference between H7N9 and Wuhan pneumonia is that H7N9 was found to be transmitted only from poultry to human beings. However, the 2019-nCoV has been found to also be transmitted from human to human [5]. The secondary cases found have indicated that there is a transmission among human beings, which can in part explain the rapid increase of the 2019-nCoV cases. The original cases could be due to transmission from animals to human beings, which is highly suspected to have happened in the Huanan wholesale seafood market. While the secondary, more rapid infections among the more recent cases could come from human to human infection. Despite the shutting down of the market, the number of new cases continues to grow. According to a predication model, the number of cases is expected to be around 19 thousand by 4 February 2020. Yet, as of 29 January 2020, there have been 5974 confirmed cases in China [6]. Hence, understanding the origin of the latest coronavirus might not be the priority in the controlling of the disease. 

Compared with Severe Acute Respiratory Syndrome in 2003, this time, the government is handling the outbreak much more quickly and transparently [7], although there is room to improve. Isolation of the possible source had been examined as an effective way to control infectious diseases. Quarantining people who have been infected has also been found to be an effective way to cut off transmission chains in infectious disease. Compared to when patients are quarantined at their home, which was applied in the case of H7N9 and others infectious diseases; transportation lock down and roadblocks are necessary in the case of Wuhan.

We propose to look back into public health’s history, back to the 19th century, when John Snow successfully traced the outbreak of Cholera in London and stopped the transmission of the pathogen. Today, we instead tend to identity the pathogen and then address how to handle and stop it. Given technological advances in science, this is often a successful and expedient way to address an outbreak of an infectious agent. However, with greater and faster movement of people around the globe, diseases can also now spread more quickly in a shorter period. As a result, public health efforts should be re-evaluated to understand what the best way is to stop the spread of pathogens. In the case of Wuhan, was a lockdown of transportation the most effective way to thwart risk of spreading the infectious agent? This is something that warrants evaluation in order to apply to future and similar situations.

At the time, when John Snow made the final decision to shut down the broad street water pump in Soho district in 1854, the people of London suspected the results would be effective, although later it was declared as the most effective measure and became the beginning of modern epidemiology. Likewise, it might be too early to evaluate the effectiveness of Wuhan’s lockdown now. Yet, there is no doubt that lockdown will greatly reduce 2019-nCoV cases due to the reduction in the secondary cases from the community. However, long-term limitations on movement of traffic and information poses a great challenge on the stability of a social system [8].

Nevertheless, it is the first time that such a national scale intervention is being conducted to stop the spread of the new emergency infectious disease. Until now, several other cities in Hubei province have also been locked down. This national innovative intervention measures enacted provides a unique experience to fight new emergency infectious diseases in the modern era.
